# The Physiological Effects of Tobacco

**Published:** 1864-12

**Authors:** W. B. Richardson


### THE PHYSIOLOGICAL EFFECTS OF TOBACCO.

By W. B. RICHARDSON, M. A., M. D.

Dr. Richardson's views on the physiological effects of tobacco were given in the following summary: 1. The effects that result from smoking are due to different agents imbibed by the smoker, viz., carbonic acid, ammonia, nicotine, a volatile empyreumatic substance, and a bitter extract. The more common effects are traceable to the carbonic acid and ammonia; the rarer and more severe to the nicotine, the empyreumatic substance, and the extract. 2. The effects produced are very transitory, the poisons finding a ready exit from the body. 3. All the evils of smoking are functional in character, and no confirmed smoker can ever be said, so long as he indulges in the habit, to be well; it does not follow, however, that he is becoming the subject of organic and fatal disease because he smokes. 4. Smoking produces disturbances: (*a*) in the blood, causing undue fluidity and change in the red corpuscles; (*b*) on the stomach, giving rise to debility, nausea, and in extreme cases sickness; (*c*) on the heart, producing debility of that organ and irregular action; (*d*) on the organs of sense, causing in the extreme degree dilatation of the pupils of the eye, confusion of vision, bright lines, luminous or cobweb specks, and long retention of images on the retina, with other and analogous symptoms affecting the ear, viz., inability clearly to define sounds, and the annoyance of a sharp ringing sound like a whistle or bell; (*e*) on the brain, suspending the waste of that organ, and oppressing it if it be duly nourished, but soothing it if it be exhausted; (*f*) on the nervous filaments and sympathetic or organic nerves, leading to deficient power in them, and to over-secretion in those surfaces—glands—over which the nerves exert a controlling force; (*g*) on the mucous membrane of the mouth, causing enlargement and soreness of the tonsils—smoker's sore throat—redness, dryness, and occasional peeling off of the membrane, and either unnatural firmness or contraction and sponginess of the gums; (*h*) on the bronchial surface of the lungs when that is already irritable, sustaining the irritation, and increasing the cough. 5. The statements to the effect that tobacco smoke causes specific diseases, such as insanity, epilepsy, St. Vitus' dance, apoplexy, organic diseases of the heart, cancer and consumption, and chronic bronchitis, have been made without any sufficient evidence or reference to facts; all such statements are devoid of truth, and can never accomplish the object which those who offer them have in view. 6. As the human body is maintained alive and in full vigor by its capacity, within certain well-defined limits, to absorb and apply oxygen; as the process of oxidation is most active and most required in those periods of life when the structures of the body are attaining their full development; and as tobacco smoke possesses the power of arresting such oxidation, the habit of smoking is most deleterious to the young, causing in them impairment of growth, premature manhoo[illegible], and physical degradation.—*Medical Times and Gazette.*

